# The Effects of Vegan Diet on Fetus and Maternal Health: A Review

**DOI:** 10.7759/cureus.47971

**Published:** 2023-10-30

**Authors:** Ogiza Palma, John Kessellie Jallah, Manjusha G Mahakalkar, Deeplata M Mendhe

**Affiliations:** 1 Biochemistry, Datta Meghe Institute of Higher Education and Research, Wardha, IND; 2 Obstetrics and Gynecology, Shrimati Radhikabai Meghe Memorial College of Nursing, Datta Meghe Institute of Medical Sciences, Wardha, IND; 3 Medicine, Community Health Nursing, Datta Meghe Institute of Medical Sciences, Wardha, IND

**Keywords:** dietary supplement, pregnant females, nutritional adequacy, fetus, vegan diet

## Abstract

Veganism, a way of eating that forbids goods produced from animals, is rising in acceptance around the globe. This thorough analysis investigates how a vegan diet affects fetal growth during pregnancy, highlighting the need to maintain ideal maternal nutrition. The idea of “early life programming” emphasizes how a pregnant woman's lifestyle impacts her unborn child's health. Nutrient consumption during pregnancy makes it essential to have a healthy eating routine. While a carefully thought-out vegan diet may contain all the essential nutrients, some micronutrients need special attention, which may call for supplementation. The study delves into significant findings concerning nutritional adequacy and challenges in the discussion section, highlighting nutrients like calcium, iron, omega-3 fatty acids, vitamin D, and protein. It emphasizes medical professionals' need to monitor and assist vegan expectant mothers in meeting their nutritional needs. The analysis also examines the intricate connection between a mother's health and the outcomes of vegan pregnancies, such as decreased rates of gestational diabetes and hypertension. Examining fetal growth and development further highlights the complexity of this process, as do the contradicting data on birth weights. Furthermore, early data suggest that infants born to vegan moms may benefit cognitively, but further studies are required to prove a causal relationship. In addressing ethical and environmental issues in the review's conclusion, it is acknowledged that these aspects impact pregnant women's food decisions. Given the rising popularity of veganism, it is crucial to offer trustworthy advice to expectant women who are thinking about or already following a vegan diet.

## Introduction and background

Veganism is a way of eating that forbids consuming products like meat, dairy, eggs, and others from animals. However, it prioritizes foods like fruits, vegetables, pulses, grains, nuts, and seeds [1]. Throughout pregnancy, it is crucial to maintain an atmosphere that is conducive to maternal health and, consequently, the development and health of the unborn children. According to the “early life” development idea, environmental circumstances and a woman's behavior, conduct, habits, styles of living, and way of acting during the carrying of a fetus affect her risk of later-life chronic illness development and impact her unborn child's future health [2]. Increased macro- and micronutrient consumption and a balanced diet are unavoidable during pregnancy. Because of this, it presents an essential period for developing dietary practices that are of a high standard for fetal health [3]. A vegan diet must be supplemented throughout pregnancy to ensure the expecting mother and the developing fetus receive all the nutrients they require for optimum health. While a well-planned vegan diet may include many essential nutrients, there are a few that pregnant women may need to pay particular attention to, and supplementation may be suggested in some cases. Data from the National Health and Nutrition Examination Survey show that between 1999 and 2014, 80.4% of pregnant women questioned used a product designated for prenatal use, and 90.8% used at least one dietary supplement [4,5]. The baby’s life from the time they are conceived until they reach two years is critical for organ development, with long-term implications for cognition, behavior, and mental and immunological health [6]. As a result, it is vital to understand which nutrients are deficient in the modern diet and how supplements might boost micronutrient consumption for pregnant women. Educating mothers about the potential effects of their diet and supplement use during this pregnancy stage is essential. Always consult with a medical expert or registered dietitian before starting any supplementation program during pregnancy to be sure it meets their unique needs. A vegan diet must be supplemented throughout pregnancy to ensure the expecting mother and the developing fetus receive all the nutrients they require for optimum health. While a well-planned vegan diet may include many essential nutrients, there are a few that pregnant women may need to pay particular attention to, and supplementation may be suggested in some cases. Always consult with a medical expert or registered dietitian before starting any supplementation program during pregnancy to be sure it meets their unique needs. To reduce potential dangers and encourage favorable fetal outcomes, we will also study ways and suggestions to maximize nutrient intake in pregnant people who adopt a vegan diet [7]. Given the rising popularity of veganism worldwide, it is essential to give pregnant women who opt for or are thinking about switching to a vegan diet during pregnancy reliable guidance.

## Review

Methodology

**Figure 1:**
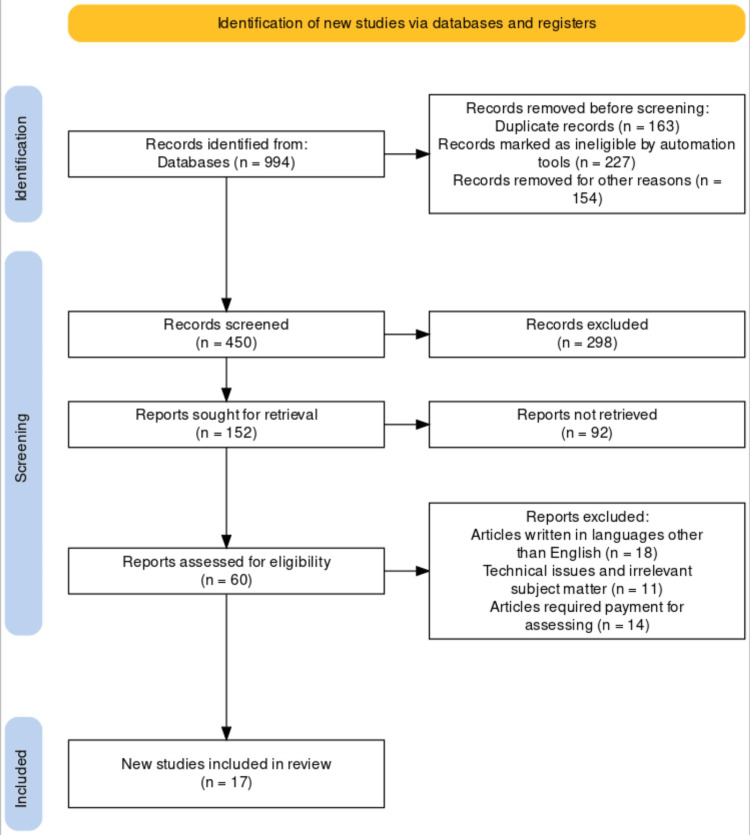
PRISMA flow diagram showing the inclusion and exclusion criteria Preferred Reporting Items for Systematic Reviews and Meta-Analyses (PRISMA)

The researchers used keywords such as vegan diet, fetus, pregnancy, nutrition, and maternal health to explore veganism's effect on the fetus's growth and development. The researchers delved into the effect of veganism on fetus health and cognitive development by conducting a comprehensive analysis through previous studies from various search engines like Google Scholar, PubMed, and Scopus using keywords such as dietary supplement, pregnant females, nutritional adequacy, fetus, and vegan diet. The following are the selection criteria included in this study, with a representation of the PRISMA flow diagram (Figure 1): (1) nutritional adequacy; (2) maternal health; (3) vegan diet; (4) fetus health and cognitive development; (5) pregnancy; and (6) English language. The following are the exclusion criteria: (1) article required payment to access; (2) article written in languages other than English; (3) irrelevant subject matter; and (4) technical issues. This review also recommends that vegan mothers routinely meet with dieticians for advice and recommended supplements to help improve both the mother's and the fetus's growth and development.

Impact of veganism during pregnancy

Within the domains of nutrition and maternal-fetal health, there is rising interest in and relevance in studying how a vegan diet affects fetal development during pregnancy. In order to offer a thorough knowledge of the possible consequences of a vegan diet on the growing baby, this in-depth review attempted to combine the body of current scientific research. It did this by referencing a wide range of studies and data sources. Expectant moms may make a healthy and moral decision by becoming vegans during pregnancy. Guaranteeing that the mother and the growing baby receive all the nutrients required for healthy growth and development, however, takes meticulous preparation. With references mentioned to support our assertions, we will go extensively into the essential results of this comprehensive debate before thoroughly examining the consequences for pregnant moms and healthcare professionals.

Nutritional adequacy and challenges

Our analysis highlights the critical significance of careful nutritional planning for expectant vegans. It is vital for individuals who follow a vegan diet to remain vigilant, even if it is widely proven that a properly designed vegan diet may supply the necessary nutrients for a healthy pregnancy. Vitamin D, calcium, iron, omega-3 fatty acids, and protein are essential nutrients that require special attention and may require supplementation to guarantee adequacy [8]. Some of the essential nutrients that are mostly obtain from animal-based sources are shown in Table 1. Healthcare professionals are essential in assisting pregnant vegan women in getting appropriate nutritional intake and avoiding deficits that can harm the development of the fetus. Comprehensive treatment for pregnant people who follow a vegan diet includes monitoring nutritional levels through routine blood testing and offering individualized dietary guidance [9].

**Table 1:** Critical nutrients, mainly obtained from animal sources, are essential for fetal development. The authors created the table illustrating the essential Nutrients, functions in Fetal Development, supplements, and Recommended Dietary Allowances for pregnant moms.

Essential Nutrients		Functions in Fetal Development	Supplement	Recommended Dietary Allowances
1	Vitamin D	Fetus skeletal development, tooth enamel formation. General fetus growth development	Vitamin D Derived from sheepwool, fish oil.	5mcg/600Iu Daily maximum=4000Iu
2	Choline	Influences brain development in fetus.	alpha-CDP-choline.	450mg
3	Calcium	Create teeth and bones. Vital for the hormones, muscles, and nerves of the developing infant.	Calcium tablets and syrup.	1000­­-1300mg
4	Iron	Increasing blood production to boost the baby's oxygen supply	Ferrous sulphate. Ferrous gluconate Ferrous fumarate.	27mg
5	Protein	Helps the baby's muscles, skin, hair, and nails grow. Supply the cellular building blocks for your child.	Protein powder	71g
6	Omega 3 fatty acid	Critical building blocks of fetal brain and retina. Early development	Fish oil supplement eicosapentaenoic acid and docosahexaenoic acid . Algae derived docosahexaenoic acid	650mg of which 300 is docosahexaenoic acid

Nutrient requirement and functions during pregnancy

Omega-3 Fatty Acid

Essential fatty acids are lipids that the body cannot produce independently and must be acquired by food or supplementation. For optimum prenatal neurodevelopment, certain essential nutrients, including docosahexaenoic acid (DHA) and eicosapentaenoic acid (EPA), can only be obtained through food [10]. The primary sources of these omega-3 fatty acids are marine foods like seafood and fish oil supplements [11]. However, alpha-linolenic acid (ALA), present in other sources of omega-3 fatty acids, including flax seed oil and vegetable oils, must be changed into the longer-chain EPA and DHA to be physiologically beneficial. Consensus standards advise pregnant mothers to ingest at least 200 mg of DHA daily to improve the quality of their pregnancies and the health of their fetuses [12,13]. According to the current recommendations of the US Food and Drug Administration (FDA) and environmental protection agency advisory, a woman can fulfill this need by consuming one to two servings of seafood per week. According to data from observational studies, consuming omega-3 fatty acids during pregnancy - either through food or as supplements - is linked to better neurodevelopmental outcomes for the unborn child [14].

Vitamin D

Most sources of vitamin D, a fat-soluble vitamin, include dietary supplements, fortified milk and juice, and fish oils. Additionally, it is generated naturally in the skin when exposed to sunlight. When vitamin D is consumed or generated by the skin, it must first be changed to 25-hydroxyvitamin D (25-OH-D) in the liver before being further converted to the physiologically active 1, 25-dihydroxyvitamin D, mainly in the kidney [15]. It takes this active form to absorb calcium from the intestines and for regular bone mineralization and development. Pregnancy-related congenital rickets, neonatal fractures, and biochemical signs of altered skeletal homeostasis have all been linked to severe maternal vitamin D deficiency [16].

Calcium

By reducing the possibility of hypertensive disorders during pregnancy, which is associated with numerous maternal fatalities and a substantial risk of preterm delivery, the leading cause of early neonatal and infant death, calcium supplementation may minimize poor gestational outcomes [17]. Contrary to PTH and calcitonin, which do not cross the placenta, the active placental transfer of substantial quantities of calcium distinguishes calcium metabolism in the fetus. Fetal calcium levels indicate that ionized calcium is considered to be conveyed from the mother to the fetus at a rate that ranges from 50 mg/day at 20 weeks to a maximum of 330 mg/day at 35 weeks of gestation [18]. Fetal parathyroid suppression and fetal calcitonin release result from the resulting fetal hypercalcemia. 25-hydroxyvitamin D passes through the placenta without any problems. However, 1, 25 (OH)2D's placental permeability is debatable. When a baby is born, the placental source of calcium abruptly ends, and the blood calcium level drops. Fetal-life-related hypoparathyroidism and hypercalcemia may make this situation worse. The neonatal calcium level reaches a minimum between 24 and 48 hours after birth, stabilizes, and then gradually increases to adult levels [19,20].

Iron

An example of a micronutrient is iron. Supporting erythropoiesis is one of iron's main nutritional functions. In the growing fetus and early child, iron, which is prioritized above all other organ systems, including the brain, is necessary for the production of hemoglobin [21,22]. Every cell and organ system in the body needs iron to form usually and then operate metabolically [23]. Its physiological purpose in iron cluster proteins and hemoproteins is to promote enzymes necessary for cellular metabolic activities, such as those that produce cellular adenosine triphosphate and provide cell oxygen. While clinical symptoms of iron deficiency anemia are linked, organ malfunction in an iron-deficient state is more likely caused by tissue-level iron shortage than by anemia. Before the emergence of anemia and even after iron therapy has resolved the anemia, signs of impaired organ function are evident [23].

Protein

Protein synthesis and the production of other nitrogenous compounds such as catecholamines, creatine, dopamine, nitric oxide (NO), polyamines, and thyroid hormones all depend on amino acids (AAs). A few AAs are also in charge of controlling metabolic and cell signaling pathways [24]. Reduced postnatal development, feed efficiency, and intrauterine growth restriction (IUGR) are all associated with low maternal dietary protein consumption [25]. The placenta requires the ideal concentration of AAs for good growth and development and to provide the fetus with the necessary nutrients. The placenta obtains insufficient AAs when the diet is inadequately protein-rich, which causes IUGR and placental insufficiency. High maternal protein consumption is also associated with IUGR and can lead to ammonia poisoning and fetal or neonatal mortality. High protein consumption during pregnancy causes AA excess, similar to low dietary protein intake [26]. 

Choline

Choline is vital for fetal development because it affects stem cell proliferation and apoptosis, which change the form and function of the brain and spinal cord and increase the risk of neural tube abnormalities and lifetime memory function [27]. The development of fatty liver (hepatosteatosis) was one of the functional effects of a dietary choline shortage in humans because phosphatidylcholine is required for the export of extra triglycerides from the liver in lipoproteins [28]. Additionally, increased serum aminotransferases were linked to liver injury in people with choline insufficiency. When liver cells were put in choline-deficient media, they died through apoptosis, which may help to explain why liver cells perished and spilled enzymes into the blood in choline-deficient patients [28,29].

Maternal health and pregnancy outcomes

Our thorough literature review sheds light on the complex link between a pregnant woman's health and the results of her pregnancy. According to research, eating a vegan diet may have benefits such as lower incidence of gestational diabetes, hypertension, and excessive pregnancy weight gain [30]. Recognizing that these outcomes need to be confirmed and that more studies are necessary to clarify the underlying processes driving any potential advantages in mother health during vegan pregnancies is crucial [31].

Fetal growth and development

When evaluating the results of pregnancies, fetal growth and development are essential considerations. According to our analysis, there is conflicting information about how a vegan diet affects embryonic growth. While some research suggests that babies with vegan moms may have smaller birth weights, other research finds no statistically significant differences [32]. It is essential to recognize that fetal development is a complicated process impacted by various variables other than maternal nutrition, such as maternal genetics, general health, and socioeconomic position [33]. Furthermore, caution is advised when interpreting these results due to the inherent difficulties in conducting dietary studies and the difficulty in accounting for confounding factors [34].

Cognitive and developmental results

Our review explores whether a mother's veganism may impact her children's cognitive and developmental results. Although these findings are still preliminary and call for more research, some preliminary studies have revealed that kids born to vegan moms may have somewhat better intelligence quotient (IQ)scores [35]. It is crucial to understand that various variables beyond maternal nutrition can significantly impact a child's cognitive development. In order to demonstrate a causal relationship between mother vegetarianism and child cognitive results, additional in-depth study is required [36].

Ethical and environmental considerations

Ethical and environmental considerations are frequently the foundation of veganism. Despite being primarily concerned with nutritional and physiological issues, our research highlights veganism's broader ethical and environmental effects during pregnancy. When choosing what to eat when pregnant, ethical issues related to animal care and environmental sustainability play a significant role. Healthcare professionals should thus discuss these moral and environmental factors with expecting moms who choose a vegan diet (Table 2).

**Table 2:** Summaries of articles used for this review.

Author’s Name	Year of Publication	Journal’s Name	Summaries
Sebastiani, et al. [2]	2019	Nutrients	During pregnancy, it is essential to create a healthy environment for the mother's well-being and the future health of the unborn child. The "early life" development concept suggests that a woman's actions, lifestyle, and behaviors during pregnancy can influence her risk of chronic illness and the health of her unborn child.
Schwarzenberg, et al. [6]	2018	Pediatrics	The first two years of a baby's life, starting from conception, are crucial for organ development and have lasting effects on cognition, behavior, and mental and immunological health.
Melina, et al. [8]	2016	Journal of the Academy of Nutrition and Dietetics	For vegans who are expecting, careful meal planning is essential. Although a well-planned vegan diet can support a healthy pregnancy, some nutrients, such as calcium, protein, iron, aomega-3 fatty, and Vitamin D, acids, may require extra care and supplementation to guarantee enough intakes.
Coletta, et al. [10]	2010	Reviews in Obstetrics and Gynecology	Essential fatty acids, like DHA and EPA, are crucial for prenatal neurodevelopment and must be obtained from food or supplements because the body can't produce them on its own.
Hibbeln, et al. [14]	2007	The Lancet	Because seafood has been linked to better neurodevelopmental outcomes for the fetus, partly because of the omega-3 fatty acids it contains, the FDA and EPA advise pregnant women to eat one to two servings of seafood each week.
Gale, et al. [16]	2008	European Journal of Clinical Nutrition	Active vitamin D is necessary for absorbing calcium from the intestines and for proper bone development. Severe maternal vitamin D deficiency can lead to pregnancy-related congenital rickets, neonatal fractures, and changes in skeletal health indicators.
Kumar, et al. [18]	2017	Journal of Obstetrics and Gynecology of India	Calcium is transported from the mother to the fetus during fetal calcium metabolism, unlike PTH and calcitonin, which do not cross the placenta. Fetal calcium levels show a rising transfer rate, starting at 50 mg/day at 5 months of pregnancy and peaking at 330 mg/day at 35 weeks.
Zamora, et al. [21]	2016	Pediatrics Research	Iron is a micronutrient crucial for supporting erythropoiesis, the production of hemoglobin. In growing fetuses and young children, iron takes priority over other organ systems, including the brain, due to its essential role in hemoglobin production.
Georgieff, et al. [23]	2019	Annual Review of Nutrition	Ferrous iron in cluster proteins and hemoproteins supports crucial enzymes for cellular metabolism, including ATP production and oxygen delivery. Iron deficiency anemia symptoms are related, but organ dysfunction in iron deficiency often arises from tissue-level iron shortage, both before and after anemia is corrected with iron therapy.
Herring, et al. [24]	2018	Experimental Biology and Medicine	Protein synthesis and production of various nitrogenous compounds, including creatine, nitric oxide (NO), dopamine, polyamines, catecholamines, and thyroid hormones, rely on amino acids (AAs). Some AAs also play a role in regulating metabolic and cell signaling pathways.
Ji, et al. [26]	2017	Journal of Animal Science and Biotechnology	The placenta needs the right amino acid levels for proper fetal development. Inadequate protein in the diet can result in IUGR and placental problems, while excessive maternal protein intake can lead to IUGR, ammonia poisoning, and fetal/neonatal mortality. Both low and high protein consumption during pregnancy can cause amino acid imbalances.
Zeisel [28]	2006	Annual Review of Nutrition	A dietary choline shortage in humans leads to fatty liver (hepatosteatosis) because choline is needed to export excess triglycerides from the liver in lipoproteins.
Tonstad, et al. [30]	2009	Diabetes Care	A comprehensive literature review reveals a complex connection between a pregnant woman's health and pregnancy outcomes. Research suggests that adopting a vegan diet may lead to reduced risks of gestational diabetes, hypertension, and excessive pregnancy weight gain.
Pistollato, et al. [32]	2015	Advances in Nutrition	Evaluating pregnancy outcomes, fetal growth and development are critical. Research on the impact of a vegan diet on embryonic growth yields conflicting results: Some studies suggest lower birth weights for babies of vegan mothers, while others find no statistically significant differences.
Meltzer, et al. [33]	2007	Maternal & Child Nutrition	Fetal development is complex and influenced by factors beyond maternal nutrition, including maternal genetics, overall health, and socioeconomic status.
Gale, et al. [35]	2007	BMJ	A review on the impact of maternal veganism on children's cognitive and developmental outcomes suggests that preliminary studies show slightly higher IQ scores in children born to vegan mothers. However, more research is needed to draw conclusive findings.
Gale, et al. [36]	2009	Journal of Child Psychology and Psychiatry	Understanding that maternal nutrition is just one of many factors influencing a child's cognitive development, further extensive research is essential to establishing a causal connection between a mom's vegetarianism and her child's cognitive outcomes.

## Conclusions

In conclusion, expectant vegans should prioritize careful nutritional planning to ensure a healthy pregnancy. While a well-planned vegan diet can provide essential nutrients, attention or supplementation is needed for protein, calcium, iron, omega-3 fatty acids, choline, and vitamin D. Medical guidance is crucial to avoid nutritional deficits that could harm the fetus. Omega-3 fatty acids, vitamin D, and calcium are vital for fetal development and maternal health. While there are potential benefits to a vegan pregnancy, further research is required to confirm the findings. Healthcare professionals should address the ethical and environmental aspects of veganism during pregnancy. Collaboration between professionals, nutritionists, and expectant mothers is essential for optimal outcomes. Wise choices and proper nutrition can lead to a healthy and successful pregnancy for pregnant vegans.
